# Dr. Edward Brown-Sequard’s Experiments and Clinical Researches—Epilepsy

**Published:** 1857-09

**Authors:** 


### SELECTIONS.

*Dr. Edward Brown-Sequard's Experimental and Clinical Researches, applied to Physiology and Pathology.*

EPILEPSY—(Continued).

A slight increase of reflex excitability is not sufficient alone for the production of epileptic seizures. It often coincides with great weakness, as is the case in old people, in convalescents, and in persons who have lost a great deal of blood. In all these cases, reflex movements take place easily under the influence of emotions, fright, or even a sudden noise. Many excitable, though healthy men and women have reflex spasms in the act of coition—hence the name given to this act by Sennert, *epilepsivbrevis.*

It is very probable that the reflex excitability, or the reflex force, of the nervous centres is extremely considerable in those persons who have fits of epilepsy for the first time, caused by a slight blow, or some ordinary moral excitement.

We shall not examine what are the parts of the cerebro-spinal axis in which there is an increase of reflex excitability, because what we have said above of the seat of epilepsy shows what are these parts, their seat being nothing but that of the increased reflex excitability, or, in other words, epilepsy consisting chiefly in that increased excitability. If it were proved that epilepsy sometimes exists only because the force of the reflex property is increased, its excitability being normal, we should have to admit that the seat of epilepsy is in almost the whole length of the cerebro-spinal axis, because, as we intend showing elsewhere, the force of the reflex property increases or decreases everywhere at the same time.

We must say, that although we admit that fits of epilepsy depend ordinarily on an increased reflex excitability, frequently combined with the existence of some special kind of irritation originating in the skin, in the mucous membranes, etc., we admit as *possible*, that without any increase of excitability, certain irritations on some parts of the encephalon may produce fits of epilepsy. We well know that the least puncture with a needle or pin of the *processus cerebelli ad pontem*, and, as I have found, of the auditory nerve, and of certain parts of the medulla oblongata, in mammals, is sufficient to produce fits of a peculiar kind of epilepsy, in which the animal rotates around the longitudinal axis of its body, in consequence of the convulsions. In man this kind of epilepsy has been frequently observed, and as the phenomena are the same as in animals (except as regards the duration of the fit, which in man is short, while in animals it lasts *usually* as long as life), it may be that the rotary convulsions have been produced, although there was no increased reflex excitability, in man as it is in animals.

Many discussions have taken place among physicians concerning the first phenomenon of a fit of epilepsy. We are, however, yet to know which of the epileptic phenomena is most frequently the first. Is it the paleness of the face, as Prof. Trousseau and others believe? Is it a spasm of the larynx, as was admitted by Dr. Marshall Hall? Or is it the loss of consciousness? We think there is no doubt that either of these phenomena may be the first, but we do not know which is most commonly the first. They may take place at the same time, and in some cases they may be entirely missing, or exist only after other phenomena.

Among the most interesting of these, ordinarily first phenomena, is the paleness of the face. Delasiauve (*Loco cit.*, pp. 56, 60, 66 and 77), considers it as extremely frequent in all kinds of attacks, from the simple slight absence of mind to the most complete epileptic seizure. Trousseau and Bland Radcliff (*London Medical Times and Gazette*, March, 1856, p. 303–304), are inclined to consider it as a constant symptom, and also as the first one. This paleness has not been explained. We consider it as a most interesting symptom, as it leads to a very probable explanation of the loss of consciousness in epilepsy. After Prof. Claude Bernard had discovered that the section of the cervical sympathetic nerve is followed by a dilation of the bloodvessels of the face, I found that when this nerve is irritated by galvanism there is a contraction of these bloodvessels, and I explained the facts discovered by the eminent French physiologist and by myself, by considering the sympathetic as the motor nerve of the bloodvessels of the face. I found, also, that the branches of the sympathetic nerve which animate the bloodvessels of the face, originate from the spinal cord with the branches of the same nerve going to the iris.—(See my *Exper. Researches in Physiol. and Pathol.* 1853, pages 9–10, and p. 75; and the *Medical Examiner*, Aug., 1852, p. 489.) The theory I then proposed has been almost universally admitted. We have in this theory an easy means of explanation of the paleness of the face in epilepsy. When the excitation takes place in the spinal chord and the basis of the encephalon, which gives rise to the fit, the nerve-fibres which go to the head are irritated, and produce a contraction of the bloodvessels. Of course this contraction expels the blood, and, in consequence, the face becomes pale. Very often another effect, depending on the nerve-fibres of the cervical sympathetic, is produced—the dilation of the pupil. But the reverse sometimes takes place—a contraction of the pupil occurring, instead of a dilation. This last phenomenon is easily explained by admitting that the excitation of the nervous centres takes place near the origin of the third and fifth pairs of nerves, and not of that of the cervical sympathetic, as is the case when the pupil dilates. The paleness of the face, and the dilation of the pupil (when it exists,) soon disappear, chiefly in consequence of the obstacle to the venous circulation in the head, and of the state of asphyxia. The cause of the obstacle to the return of blood from the head is not only the contraction of the muscles of the neck, as Dr. Marshall Hall seems to think, but also in the state of the chest. Dr. Russell Reynolds (*Diagnosis of Diseases of the Brain, etc.*, 1855, p. 176,) says that he has observed in many cases in which the muscles of the neck were quite flaccid, notwithstanding the darkness of the face, and the leaden hue of the body generally.

Among one of the first symptoms of the fit, and as a cause of the cry, there is a spasm of the laryngeal muscles, and a contraction of the expiratory muscles. This contracted state of the chest acts on the heart so as to diminish the force of its beatings, as is the case in the experiment of compressing the chest, made by E. Webber and others, and it acts on the veins, in preventing the circulation in them. Although compressed, and unable to beat freely, the heart quickly recovers an apparently great strength; the blood, losing its oxygen and becoming black, acts as a powerful irritant upon the central organ of circulation, so that palpitations, sometimes very violent, occur. Nevertheless, the pulse often remains weak, because the quantity of blood sent to the arteries by the heart is smaller than usual, on account of the obstacle to venous circulation.

We think that at nearly the same time, when the origin of the branches of the sympathetic nerve going to the bloodvessels of the face receive an irritation in the beginning of a fit of epilepsy, the origin of the branches of the same and of other nerves, going to the bloodvessels of the brain proper, also receive an irritation. A contraction then occurs in these bloodvessels, and particularly in the small arteries. This contraction expelling the blood, the brain proper loses at once its functions, just as it does in a complete syncope. Now, as it has been well proved by the researches of Kellie, of Abercrombie, of John Reid, of Henle and of Foltz, that the quantity of liquid in the cranio-spinal cavity cannot change suddenly, it results, that if there is less blood in tho brain proper there must be more in the basis of the encephalon and in the spinal cord. In consequence of the impediment of respiration, the blood sent to the encephalon, as well as to other parts of the body, contains but little oxygen, and is charged with carbonic acid, so that the large quantity of blood accumulated in the basis of the encephalon (the medulla oblongata, the pons Varolii, the tubercular quadrigemina, etc.,) and in the spinal cord, is endowed in a high degree with the power which I have shown that such blood posesses, *i. e.*, to excite convulsions. It may be as Henle has supposed, that the basis of the encephalon is then excited to cause convulsions in consequence of the pressure exerted upon it by the accumulation of blood. The spinal cord, also, in all its length, is then excited to produce convulsions by the blood which circulates in it. The grounds on which I base these views are the following.

1st. There is, in the beginning of a complete fit of epilepsy, an irritation of the parts of the nervous centres from which originate the nerve-fibres of the bloodvessels of the brain, and therefore there ought to be a contraction of these vessels. The cervical sympathetic nerve contains not only the nerve-fibres which cause a dilation of the pupil, and those which produce the contraction of the bloodvessels of the face in the beginning of a fit, but also the nerve-fibres of the bloodvessels of the brain.—Prof. Claude Bernard (*Memoires de la Soc. de Biologie*, for

1853, p. 94) has found that when the cervical sympathetic nerve is divided on one side, the temperature of the brain is increased in the corresponding side. We have shown that this elevation of temperature depends on the circulation of a larger amount of blood, which is the consequence of the paralysis of the bloodvessels, due to the section of their nerve-fibres. Some experiments of Donders, and his pupil, Van der Beke Callenfells, have also shown the influence of the sympathetic on the arteries of the pia mater (see Donder's *Physiologie des Menschen* Leipsic, 1856, p. 138 and 140;) they have seen these arteries contract when the sympathetic was irritated.

2d. We have said that we consider as reflex the convulsions of epilepsy, whether they depend on centric or excentric excitations. The contraction of the bloodvessels of the brain and face in a fit of epilepsy are also reflex. We have proved elsewhere (see my *Exper. Researches in Physiol. and Pathol.*, 1853, p. 34) that bloodvessels may contract by a reflex action, as well as muscles. In experiments with our distinguished friend Dr. Tholozan, Professor at the Military Medical School of Paris, we have found that the bloodvessels of one hand contract, by a reflex action, when the sensitive nerves of the other hand are irritated by being exposed to the influence of water at the freezing point. Schiff (*Comptes Rendus de l'Acad. des Sciences*, vol. xxxix., 1854, p. 509.) Donders (*loc cit.*, p. 139,) myself and more recently M. Vulpian (*Gaz. Med. de Paris*, 1857, p. 18,) have found that the bloodvessels of the ear in rabbits contract by reflex action, when the central part of the divided auricular nerve is irritated. I have found, besides, that the splanchnic nerves and other branches of the sympathetic have a reflex action of the bloodvessels of the heart. (See my paper, *Recherches Experim. sur la Physiol. et la Pathol. des Capsules surrenales*, 1856, p. 30.) All these facts establish beyond doubt that the bloodvessels as well as muscles of animal life may contract by a reflex action. In a seizure of epilepsy, therefore, the bloodvessels of the brain proper, those of the face, the muscles of the neck, of the larynx, etc., may contract by a reflex action, either separately or at the same time.

3d. To say that an explanation is a good one because we do not know or because we cannot imagine any other one, is an argument which rarely has any value; but when the explanation is not only possible, but also rendered very probable, as is the case with our theory of the loss of consciousness in epilepsy, it is an argument of a positive value that no other theory, except one or two having many facts against them, has been proposed heretofore.

4th. It might be objected to the explanation we propose, that the loss of consciousness is too rapid to be due to a contraction of bloodvessels. There is a fact which answers peremptorily this objection; it is, that when the cervical sympathetic is irritated by a powerful electro-magnetic current, the contraction of the bloodvessels of the face, and particularly of those of the ear, is almost immediate, and so considerable that many of the small arteries seem to expel completely their contents. Now, as everybody knows that even a diminution in the supply of blood to the head, as in ordinary syncope, is sufficient to produce an immediate loss of consciousness, *a fortiori* is it so if the nerve-fibres, irritated in the nervous centres, produce a contraction in the bloodvessels of the brain proper.

5th. As we see that the bloodvessels of the face, after a contraction of very short duration, dilate and become turgid, it might be asked if it be not so with the bloodvessels of the brain proper, and why, in that case there is no return of consciousness when the blood returns in the dilating bloodvessels. We answer that it is probable that the cerebral bloodvessels dilate, like those of the face; but that when this dilation takes place, the blood which then reaches the brain does not contain oxygen enough, and is charged with too much carbonic acid, to be able to regenerate the lost function of this organ. It is only when the respiration has become almost completely free, that the functions of the brain re-appear.

6th. It might be objected, also, that the theory does not explain why the nerve-fibres going to the bloodvessels of the brain proper are excited, while those of the bloodvessels of the base of the encephalon are not. The theory has not to explain this difference; *it is a fact* that the action of the brain proper i lost, while the action of the basis of the encephalon is very much increase d, during a fit of epilepsy; and all that the theory has to do is to explain the loss of action in one part, and the cause of increased action in another. However, we may add that if the bloodvessels of the base of the encephalon are not excited to contract, it is, according to all probability, because their nerves originate in another place from those of the cerebral bloodvessels; and as we know that the nerves going to certain muscles are excited in the beginning of a fit, while others are not, we may understand easily that the same thing exists for the nerves of the various encephalic bloodvessels.

7th. As regards the influence of blood charged with carbonic acid on the nervous centres, we will refer to our often-quoted work (p. 80, and p. 101–124;) and we will merely say here that we have found that the injection of blood charged with carbonic acid into the carotid or into the vertebral arteries, at once causes epileptiform convulsions.

8th. It might be objected that the bloodvessels of the base of the encephalon and of the spinal cord ought to be excited to contract by two causes after the fit has lasted some time; the first cause being the excitation of all the parts of the cerebro-spinal axis in which there is blood charged with carbonic acid, and, consequently, the excitation of the nerves of the blood-vessels of the basis of the encephalon, because these nerves take their origin somewhere in those excited parts of the cerebro-spinal axis; the second cause being the direct excitation of the smooth muscular fibres of the bloodvessels of the encephalon by the blood charged with carbonic acid. Now if the blood-vessels contract, whether it is on account of the first or of the second cause, or of both, it seems that the fit ought to be diminished at once. But in the first place, it is probable that the bloodvessels contract irregularly, some at one time, some at another. In the second place, blood charged with carbonic acid, after its first action (which is an excitation) has a secondary action, which causes the loss of the contractility of the muscular layer of the bloodvessels. In the third place, the obstacle to the return of venous blood may cause the bloodvessels to dilate to such an extent that they cannot contract, as is the case with the heart when its cavities are too full.

